# Norwegian health personnel’s contacts and referrals for children of ill parents: an exploratory cross-sectional multi-centre study

**DOI:** 10.1186/s12913-023-09607-0

**Published:** 2023-06-17

**Authors:** Kristin Stavnes, Torleif Ruud, Jūratė Šaltytė Benth, Ketil Hanssen-Bauer, Tytti Solantaus, Marit Hilsen, Bjørg Eva Skogøy, Ellen Katrine Kallander, Elin Kufås, Bente M. Weimand

**Affiliations:** 1 The Regional Centre for Eating Disorders (RESSP), Nordland Hospital Trust, 8092 Bodø, Norway; 2 Faculty of Medicine, University of Oslo, Oslo, Norway; 3 The Regional Centre for Eating Disorders (RESSP), Nordland Hospital Trust, Kløveråsveien 1, 8076 Bodø, Norway; 4 Division of Mental Health Services, Akershus University Hospital, Lørenskog, Norway; 5 Institute of Clinical Medicine, University of Oslo, Campus Ahus, Akershus, Norway; 6 Health Services Research Unit, Akershus University Hospital, Lørenskog, Norway; 7 Department of Public Health and Welfare, Finnish Institute for Health and Welfare, Helsinki, Finland; 8 Regional Centre for Child and Adolescent Mental Health, RBUP Øst Og Sør, Postboks 4623, 0405 Nydalen, Oslo Norway; 9 Nordland Research Institute, Postboks 1490, 8049 Bodø, Norway; 10 Vestre Viken Hospital Trust, Drammen, Norway; 11 Department of Health, Social and Welfare Studies, Faculty of Health and Social Sciences, University of South-Eastern Norway, Drammen, Norway

**Keywords:** Legislation, Law, The Act, Referrals, Children of ill parents, Parental illness, Mentally ill parents, Parents with substance abuse, Physically ill parents, Somatically ill parents

## Abstract

**Background:**

In 2010, changes were made to the Norwegian Health Personnel Act. This led to all health personnel being obliged to support the patients’ children and families. The aims of this study were to investigate whether health personnel contacted or referred the patients’ children to family/friends or public services. We also investigated if there were factors in the family or the services that increased or decreased the degree of contacts and referrals. In addition the patients were asked whether the law had been a help or even a burden. This study was part of a larger multi-site study of children of ill parents conducted in five health trusts in Norway.

**Method:**

We used cross-sectional data from 518 patients and 278 health personnel. The informants completed a questionnaire addressing the law. Data were analyzed by factor analysis and logistic regression.

**Results:**

The health personnel contacted/referred children to different services, but not to the degree desired by their parents. Only a few contacted family/friends, or the school and/or the public health nurse, those representing the helpers who live closest to the child, and thus well situated to participate in help and preventive efforts. The service most often referred to was the child welfare service.

**Conclusion:**

The results indicate a change in contacts/referrals for children from their parents’ health personnel but also reveal remaining needs for support/help for these children. Health personnel should strive to write more referrals and take more contacts than the current study suggests, to secure adequate support for children of ill parents in Norway, as intended in The Health Personnel Act.

## Background

In 2010 the Norwegian Parliament approved an amendment to the Health Personnel Act, § 10–4, hereafter referred to as The Act. All health personnel were required to assure that children of ill parents (CHIP) shall receive needed information and support [1]. The Act applies to all mental health, substance use and physical health services, although only severe physical illness is included, and it applies to all levels of health care, from municipal services to specialized health services [1]. Health personnel are obliged to ask patients whether they have children younger than 18 years, and, if so, to have a conversation with them about their parental capacity, their children’s situation and their needs for information about their parent’s situation. Children should be invited to visit the parent during episodes of care. If needed, the other parent and the child/children shall be invited to a conversation at the hospital, where the child/children should be given age-appropriate information, and if necessary, be referred to further help or support. This could be informal contacts, hereby defined as telephone calls, mails or direct contact to friends, family or public services, or formal written referrals to a range of municipal services or specialist health services. Hospitals are required to appoint child-responsible personnel to coordinate this work. Similar legislation exists in the other Scandinavian countries [2–4]. This paper will focus on the last part of The Act: The contacts/referrals of the CHIP to appropriate persons/services.

The intention of The Act is to prevent negative consequences for CHIP and ensure that these children receive support to go on with their normal lives even when a parent is ill. In the event that a child already suffers from mental health problems, health personnel shall ensure that he or she receives appropriate treatment. For more than two decades, it has been well-documented that CHIP have an elevated risk of mental illness regardless of whether the parent suffers from physical or mental disorders or substance use disorder [5–16].

A few studies have compared risk in regard to the three health service domains mentioned above. They have identified a tendency for children of mentally ill parents and, especially, parents with substance use to be somewhat more likely to suffer from mental health problems than children of physically ill parents [17–20]. One study indicated that children of parents with combined substance use problems and mental illness are at greater risk than children with parents who have one of these problems [21]. We found no studies that compared referrals of children in regard to the different health service domains.

In most countries the primary health care with general practitioners, the school system and public health nurses are responsible for referring children to child welfare services and specialist health services [22, 23]. The current Norwegian legislation requires health personnel to make contacts/referrals in the reverse direction as well, that is, from the specialist health care system to primary health care and from adult health services to children’s health services. Several studies have noted that this is necessary, although difficult, and have pointed to ‘silos’ (that those services do not cooperate) as a problem in the health/support system [24–27].

In Norway no baseline data regarding numbers or proportions of contacts/referrals for CHIP prior to the implementation of The Act existed at the time of the present study, but some information is available. Three reports from the three health domains found that CHIP were lacking support to a substantial degree [28–30]. They highlighted a lack of collaboration between health care services for adults and children as well as between the municipal services and the specialist health services. Others have reported similar findings [25]. Two recent quantitative Norwegian studies found that general practitioners engage in situations related to CHIP only to a small extent, even following the enactment of the legislation [23, 31].

Moreover, implementation strategies following the law’s adoption were lacking. Some stakeholders were prepared, but in general, economic recourses, leader involvement, education and data systems were not in place at the time The Act was introduced. Child-responsible personnel were appointed only to a limited degree [32–34].

Health care professionals and patients tend to have different views on illness, treatment, and also on what has happened during a hospital stay [35–37]. Thus, including both informant groups could provide a more nuanced picture.

We wanted to investigate whether some family and service factors could predict whether or not patients’ health personnel would contact or make referrals to the relevant persons and/or services. One factor was the children’s age. An Australian review of programs for CHIP reported that only three out of 20 programs included children under eight years old, which could indicate that younger children are under-prioritized [38]. A meta-analysis by Sieh et al. of children of physically ill parents found that the youngest children and adolescent girls of ill mothers were at the highest risk level for maladjustment [6].

Level of education has been shown to be positively associated with information-seeking in medical consultations [6]. We did not find that this also implied requests from parents for referrals regarding their children. Only one study examined the effect of duration of patients’ illness on CHIP. Sieh et al. identified larger negative effects for children of parents with the longest illness duration [6]. To our knowledge, no studies have explored either the association between referrals and income or between referrals and duration of illness. Nor did any studies compare referrals for CHIP in the different health service domains.

A large-scale prevention effort should, primarily, have positive consequences, and whether or not the program is perceived helpful to the participants should be explored [39].

Based on the above, we posed the following research questions:

### Research questions

- 1) To what degree were contacts/referrals made on behalf of CHIP, to which services were contacts/referrals made, and were these contacts/referrals in accordance with the ill parents’ expectations and wishes?
- 2) Were contacts/referrals associated with characteristics of the family and the services, i.e. children’s ages and mental health statuses, patients’ levels of education, income, patients’ symptoms, and types and durations of illness?
- 3) Did patients experience The Act as helpful or even as a burden?

## Method

### Design

This study was part of a Norwegian cross-sectional, multi-centre, quantitative CHIP study conducted in five health trusts (specialist health services) in Norway three to four years after The Act came into force [40]. We chose the cross-sectional design for making a fast and broad picture of the implementation-process of The Act in Norway [41]. The participating health trusts cover 34% of the Norwegian population and include physical, mental and substance use health services. The overall objective of the main study was to explore the situation among CHIP and their families. The present study used a subset of these data to examine contacts/referrals made on behalf of the children.

### Recruitment and procedure

Families were recruited through the ill parents who were being treated by specialist health services in physical health, mental health and/or substance use services. Recruiters visited units in physical health, mental health and substance use health, in out- and inpatient services on randomly selected days. Health personnel were encouraged to ask all patients with children 0–18 years of age whether a study coordinator could give them information about the study. If the patients agreed to be informed, verbal and written information was provided. Recruiters were researchers or trained clinicians. The inclusion rate of eligible families is unknown, as it was not possible to receive reliable data on the number of patients who were informed about the study and invited to participate. Clinicians, especially in outpatient clinics, may have forgotten to inform the patients, and some stated that they were reluctant to inform their patients, eg. if they believed he or she was too ill to participate.

The patients were eligible if they had regular contact (at least every other week) with their children and could read Norwegian. If a patient had more than one minor child, we arbitrarily chose one of them. If, for any reason, the parent did not want this child to be included, we randomly selected another child. If the patient consented, we asked the responsible health personnel to participate. All health personnel and patients that agreed to participate were included in the study.

The researchers/co-workers afterwards met the patients according to their wishes. The majority wanted to meet at their home in the afternoon. They answered questions in a de-identified manner using tablets linked to a database. Researchers assisted them in the event of any content or technical problems. The health personnel received a link to a website the informants responded to. The data were collected from May 2013 until the December 2014.

### Participants

**Fig. 1:**
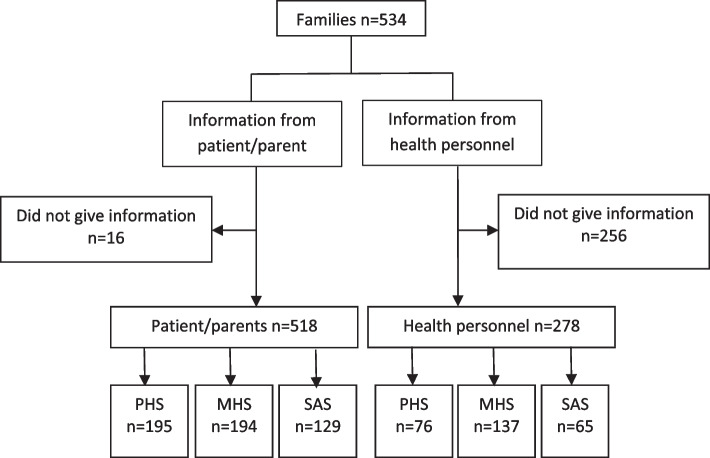
Flowchart of participating respondents. Abbreviations: *PHS* Physical health services, *MHS* Mental health services, *SAS* Substance abuse services

The study sample comprised 518 patients and 278 health personnel. The patients were from either physical health (*N* = 195), mental health (*N *= 194) or substance use services (*N* = 129). Since the legislation applies only to severe physical illness, we included only those patients with cancer or severe neurological diseases from physical health services. Health personnel participating included nurses (35.3%), psychologists (32.4%), physicians (16.2%), social workers (5.0%) and others (11.2%). Among them, 76 represented physical health, 137 mental health and 65 substance use services. Figure 1 shows a flow chart of the participating informants.

Of the patients, 358 were mothers (69.1%) and 160 fathers (30.9%). Mean age was 38.1 years, and most, 483 (93.2%), were ethnic Norwegians. The majority, 326 (62.9%), were married or lived with a partner. Patients were biological parents for 272 (52.5%) of the children and cared for an average of 2.1 children.

The educational level of patients was slightly above the average national level; 102 (16.7%) had finished elementary school; 221 (42.7%) had completed high school; and 195 (37.7%) had studied at a college or university. In regard to work, 156 patients (30.1%) worked full time, 80 (15.4%) part time and 39 (7.5%) were students; 127 (24.5%) were temporarily on sick leave, which in Norway is paid by the state. In summary, 240 patients (46.3%) received other kinds of permanent economic support from the state. Mean income for the households was 723,588 Norwegian kroner, somewhat below the average in Norway [42]. Sociodemographic characteristics were reported by the patients. Please see Table 1 for more details.

**Table 1:** Descriptive statistics of child, patient and family characteristics

Characteristic	Children 8 − 18 years	Patients (with children 0–18 years)	Health personnel (reporting on children 0–18 years)
Childs age in years, mean (SD)	12.5 (2.9)	8.3 (4.8)	7.9 (4.7)
Education patient, n (%)			
*Elementary school*		102 (19.7)	
*High school*		221 (42.7)	
*College/university*		195 (37.6)	
Income household in NOK, mean (SD)		727,270 (387,368)	
Patient’s physical health score, mean (SD)		45.1 (10.6)	
Patient’s mental symptoms, mean (SD)		20.3 (7.5)	
How long child has known about parent’s illness			
*Got to know recently, n (%)*	30 (12.2)		
*Several months, n (%)*	90 (36.6)		
*Several years, n (%)*	86 (35.0)		
*Have always known, n (%)*	40 (16.3)		
Duration of patient’s illness in years, mean (SD)		8.3 (8.9)	9.2 (8.7)

### Measures

All the participating informants were able to fill out the measures. Sociodemographic characteristics were collected as part of the electronic questionnaire the participants filled in, and the results are reported in Table 1. As we did not find any validated questionnaires that measured the constructs relevant to this paper and to The Act, experienced researchers and clinicians designed a new measure with questions aligned to the formulations of The Act. The questions relevant to this paper are presented in Table 2, where numbers and percentages of those answering ‘yes’ are reported.

**Table 2:** Frequencies and percentages that confirmed that the children/families had been followed up on the HPs obligations in the Act

	Patients (*n* = 518)	Health personnel (*n* = 278)
	n (%)	n (%)
Did the health personnel contact friends/family to attend to the needs of the child?	53 (10.2)	28 (10.1)
Did the health personnel contact municipal health/social service, to attend to the needs of the child?	74 (14.3)	56 (20.1)
Did written reports to the GP after the hospital’s treatment say anything about the child’s needs?	16 (3.1)	62 (22.3)
Did the health personnel refer the child to appropriateservices?	108 (20.8)	29 (10.4)

Physical health was measured using four questions from the Short Form Health Survey (SF-8) [43], which is a validated and frequently used instrument [43–45]. The questions, which have five or six response alternatives, are asked in reference to the previous week. The higher the score, the better the respondent’s quality of life related to his or her physical health. In our study Cronbach’s alpha was 0.80.

Mental health was measured by the Hopkins Symptom Checklist (HSCL-10), a short version of the HSCL-90 developed by Derogatis [46]. The HSCL-10 is a validated and widely used instrument [47, 48]. Four questions pertain to anxiety and six to depression. A higher score indicates greater symptomatology. In our study Cronbach’s alpha was 0.92.

The Strength and Difficulties Questionnaire (SDQ) measures children’s mental health. It has been widely used and has shown good psychometric properties [49, 50]. It comprises five subscales: emotional symptoms, conduct problems, hyperactivity/inattention, peer relationships, and prosocial behavioral. Eight additional questions refer to the degree of consequences of the child’s problems. A higher score indicates a larger number of mental difficulties.

### Statistical analysis

#### Contacts and referrals were presented as frequencies and percentages

A factor analysis with principal component extraction method and varimax rotation of the questions about contacts and referrals was conducted separately for patients and health personnel, for those families where both the patient and the health personnel participated. Ordinal variables were dichotomized for factor analysis, and correlations were used as input. All questions were assessed in the analysis, but only relevant factors were further explored in this study. Factor scores were dichotomized to ‘yes’ and ‘no’ based on logical assessment of the variables loading on the relevant factor [51].

Unadjusted and adjusted logistic regression analyses were performed to assess the associations between dichotomized factor scores (the dependent variables on how health personnel contributed to referrals/contacts for children) and children’s ages, parents’ education and income, severity of parents’ illness, duration of patients’ symptoms and the service to which the patients belonged. All health personnel and patients were included in these analyses.

All tests were two-sided. Results with *p*-values below 0.05 were considered statistically significant. All data analyses were performed in SPSS v24 [52].

The study had no missing data due to the data program, which did not allow informants to continue to the next question until the present question had been answered. However, as we allowed answers such as ‘I do not know’ and ‘not applicable’, we chose to treat these as missing values in all but descriptive analyses. Whenever possible, we handled missing values through logic imputation.

### Ethical approval

This study was approved by the Regional Committee on Medical and Health Research Ethics South-East (reg. no. 2012/1176) and by the Privacy Ombudsman at each of the five health trusts participating in the study. All informants gave their informed written consent.

## Results

### Contacts on behalf of the children

According to Table 2, health personnel and patients reported that about 10% of the health personnel did contact friends and family on behalf of the child. They reported that health personnel had mentioned the patients’ children to the municipal health/social services to a greater extent. While 22.3% of the health personnel stated that their patient’s children were included in the written reports to the patients’ GP from the patients’ treatment in specialist health services, only 3.1% of the patients confirmed that this was the case.

### Referrals of the children

According to Table 2, the health personnel responded that they had formally referred 10.4% of their patients’ children to appropriate municipal services (e.g. the school, GP, public health nurse, child and adolescent mental health service or child welfare services) while the patients responded that this applied for 20.8% of the children. Another 40.0% of the patients reported that they wished their children had been referred to at least one of these services, see Table 3.

**Table 3:** Frequencies and percentages of referrals to different services

	Health personnel (*n* = 278)	Patients’ assessment (*n* = 518)	Patients’ wish (*n* = 518)
To which service was the child referred?	n (%)	n (%)	n (%)
Extra help from the school / teacher / kindergarten	4 (1,4)	28 (5,4)	114 (22,0)
The child’s GP		0 (0,0)	29 (5.6)
The community nurse	8 (2,9)	32 (6,2)	78 (15,1)
Child and adolescent mental health services	10 (3,6)	17 (3,3)	53 (10,2)
Pedagogic psychological services in schools	0 (0,0)	13 (2,5)	31 (6,0)
The child welfare service	14 (5,0)	45 (8,7)	21 (4,1)
Group for children / adolescents who live with ill parents	2 (0,7)	9 (1,7)	37 (7,1)
Others	4 (1,4)	25 (4,8)	31 (6,0)

The health personnel referred the children to services as shown in Table 3. While the patients primarily wanted the health personnel to refer their children for additional follow-up by the school system and the public health nurse, the health personnel most often referred the children to the child welfare service (5%). The health personnel in the substance use services even more often referred the child to this service (19% of the children, a result not referred to in any table).

### Factors correlated with contacts/referrals

Questions related to contacts/referrals loaded on one factor for the patients and one factor for the health personnel, with Cronbach’s alpha 0.69 and 0.65 respectively. Factor loadings are presented in Table 4.

**Table 4:** Results of factor analysis

Item	F1	F2	F3	F4	F5	F6	F7	F8
***Patients***								
Did HP contact services in the municipality, in relation to the child’s needs for information and help?	0.19	**0.79**	0.19	-0.00	0.08	0.04	-0.02	
Was the child referred to any services?	0.07	**0.73**	0.08	0.03	0.07	-0.08	-0.12	
Did HP contact friends/family to be aware of the child’s needs?	0.09	**0.73**	0.01	0.03	0.14	0.15	0.13	
Did the discharge letter after treatment contain information about your child's needs?	-0.02	**0.51**	0.51	-0.10	-0.02	0.06	0.07	
*% of total variance explained*		9.9						
*Cronbach’s alpha*		0.69						
***HP***								
Did you contact services in the municipality, in relation to the child’s needs?	-0.02	**0.74**	0.05	0.02	-0.00	0.15	0.02	-0.06
Did the discharge letter or other documents contain information about the child?	0.01	**0.64**	0.14	0.16	0.15	0.16	-0.05	-0.05
Did you refer the child to any service during the patient’s treatment?	-0.20	**0.60**	0.25	-0.02	-0.14	-0.08	0.20	0.00
Did you contact friends/family to be aware of the child’s needs?	0.14	**0.59**	0.15	0.18	-0.01	-0.03	0.13	-0.22
*% of total variance explained*		12.6						
*Cronbach’s alpha*		0.65						

According to adjusted logistic regression analysis for patients, more-pronounced children’s mental symptomatology (a higher SDQ score) and a parent being in substance use services compared to physical health services were associated with a higher likelihood of children being referred (Table 5).

**Table 5:** Results of unadjusted and adjusted logistic regression analysis

***Patients***				
Characteristic	Unadjusted models		Adjusted models	
	OR (95% CI)	*p*-value	OR (95% CI)	*p*-value
Child’s age	1.00 (0.99; 1.01)	0.514	1.01 (0.99; 1.01)	0.135
Education patient				
Elementary school	2.02 (0.94; 4.36)	0.073	1.32 (0.57; 3.07)	0.520
High school	2.32 (1.27; 3.92)	**0.005**	1.50 (0.81; 2.79)	0.198
College/university	1	-	1	-
Income household^a^	0.90 (0.84; 0.98)	**0.007**	0.98 (0.90; 1.06)	0.618
Patient’s physical symptoms	0.98 (0.95; 0.99)	**0.040**	0.97 (0.95; 1.00)	0.051
Patient’s mental symptoms	1.07 (1.04; 1.11)	** < 0.001**	1.04 (0.99; 1.08)	0.089
Duration of illness^b^	1.01 (0.99; 1.04)	0.190	0.99 (0.97; 1.02)	0.618
Part of health care system				
Physical health services	1	-	1	-
Mental health services	2.49 (1.38; 4.47)	**0.002**	1.92 (0.89; 4.15)	0.099
Substance use services	2.61 (1.32; 5.18)	**0.006**	3.15 (1.26; 7.90)	**0.014**
SDQ according to patients	1.10 (1.06; 1.16)	** < 0.001**	1.08 (1.03; 1.14)	**0.003**
***HP***				
Characteristic	Unadjusted models		Adjusted model	
	OR (95% CI)	*p*-value	OR (95% CI)	*p*-value
Child’s age	1.00 (0.99; 1.01)	0.552	1.01 (0.99; 1.01)	0.917
Education patient				
Elementary school	2.10 (0.79; 5.58)	0.137	1.40 (0.45; 4.36)	0.281
High school	1.65 (0.73; 3.77)	0.232	1.23 (0.51; 3.01)	0.267
College/university	1	-	1	-
Income household^a^	0.88 (0.78; 0.99)	**0.018**	0.94 (0.82; 1.07)	0.933
Patient’s physical symptoms	0.97 (0.94; 0.99)	**0.041**	0.96 (0.92; 0.99)	0.394
Patient’s mental symptoms	1.06 (1.02; 1.11)	**0.008**	1.03 (0.97; 1.09)	0.675
Duration of illness^b^	1.01 (0.97; 1.05)	0.421	0.99 (0.95; 1.03)	0.970
Part of health care system				
Physical health services	1	-	1	-
Mental health services	4.05 (1.32; 12.43)	**0.014**	4.7 (1.2; 18.0)	**0.023**
Substance use services	4.69 (1.41; 15.59)	**0.012**	7.0 (1.5; 33.0)	**0.013**

^a^Per 100,000-change, ^b^Per 10-change

Regarding health personnel, both the mental health and the substance use services were associated with a higher likelihood of contacts/referrals compared to the physical health services.

The children’s ages, patients’ education level and income, and the severity and duration of their illness did not influence the odds for contacts/referrals conducted by the health personnel.

### The helpfulness of the legislation

Table 6 shows that only a minority of patients were explicitly informed about the new legislation by the health personnel. There were small differences regarding whether the patients reported that the legislation was helpful, respectively 42.6%, 37.1% and 37.2% among the physical health, mental health and substance use service users, see Table 6. However, we found a large difference when asking patients whether it had been difficult for them to have the health personnel focus on their child’s situation. While only a few patients from the physical health and mental health services confirmed this, 29.5% from the substance use services confirmed it.

**Table 6:** Frequencies and percentages of satisfaction with the legislation_

	Physical health patients *n* = 195	Mental health patients *n* = 194	Substance use patients *n* = 129	All patients *n* = 518
	n (%)	n (%)	n (%)	n (%)
Did the health personnel inform you about the legislation?	74 (37.9)	78 (40.2)	57 (44.2)	209 (40.3)
Has the legislation been of help for the child and the family?	83 (42.6)	72 (37.1)	48 (37.2)	203 (39.2)
Has the legislation been a burden?	10 (5.2)	16 (8.3)	38 (29.5)	64 (12.4)

## Discussion

### Contacts on behalf of the children

The health personnel reported that they contacted public services on behalf of the patients and families more often than they contacted the patients’ network of friends and family members. This may indicate a potentially unused resource among the patients’ network of friends and family. Studies of meetings with friends and family members show encouraging results and could probably be used more often. Furthermore, such networks often want to contribute, but they may be uncertain about how to go about doing so. A Norwegian research team that has studied these challenges and opportunities has identified ways for these networks to help. They found that family and friends can be valuable helpers [53–55].

The patients reported that health personnel had contacted friends/family and local services twice as often as the health personnel themselves reported contacting these sources. Moreover, the health personnel reported more often than did the patients that the children were mentioned in the discharge letters to the GP. This could indicate some ambiguity in the communication between patients and health personnel, i.e. that health personnel did not inform their patients properly on what they did for their patients’ children. Previous research has reported similar results [35–37]. Disappointments may result when people have different expectations. This emphasizes the importance of securing a mutual understanding between patients and health personnel, indicating health personnel’s need for communication skills. Another Norwegian study reported likewise [56]. Another reason may be the quite large differences in response rates between patients and health personnel.

### Referrals of the children

Among the health personnel, 10% reported that they had referred patients’ children to other services, while 20% of the patients reported that this had been done and 40% of the patients wanted referrals for their children. Again, this may indicate communication problems between patients and their health personnel.

Our numbers indicate that a minority of the children who needed help actually received it, which is in line with other research from child and adolescence mental health [16, 35–37]. A Norwegian study confirm that even when CHIP are identified, they may not be referred to appropriate services [57]. Another cross-sectional Norwegian study, where 23,167 outpatients in mental health services participated, found that 36% of these patients’ children were referred to a service, and that 58% of them were considered not having needs for that [58].

These findings are in accordance with a 2006 review that concluded that only a few children who were experiencing mental health problems received help [59]. The review found that one-third of the children with mental health needs were receiving treatment, and that the parents’ assessment of their children’s needs was a key to the first step in help-seeking. Another review from primary health care identified a broad range of barriers related to identification, management and/or referrals, including lack of providers and resources, waiting lists, and financial restrictions [60]. Likewise, Landeweer et al. in their review found that the ill parent, the professionals, the organization of care and the culture-paradigm could all be barriers to family involvement when a parent is ill [61]. A review from the mental health field disclosed a number of barriers to securing help for the patients’ children, such as not recognizing whether the patients are parents, not having adequate policies and procedures, and lack of competence in regard to families, children and parenting [25]. An implementation study, conducted as part of the CHIP study, pointed to organizational and professional factors and appointing of child-responsible personnel as important for the implementation of the new efforts in health services [62].

Even if not sufficient, our findings indicate a positive change in whether health personnel refer their patients’ children and how they understand the needs of these children. This is in accordance with recommendations from several professionals in this field [8, 25, 26, 63, 64].

The informants differed in regard to the services to which they reported having referred the children. According to the health personnel, they referred the children primarily to the child welfare service and, thereafter, to child and adolescent mental health service and the public health nurse. This is in line with another Norwegian study of adult mental health outpatients [58] where patients reported that most of their children were referred to the child welfare service, and thereafter to the public health nurse and the school. In particular, parents’ wishes regarding referrals to the school and the public health nurse were not in agreement with the reports from the health personnel in this study. The health care system in Norway and many other countries is arranged to provide assistance at the lowest possible level. It could be suggested that for CHIP, it is most appropriate to receive support from people they are close to and at a local level, such as family members and friends, schools and community nurses. Helpers who know the children well might increase the possibility of providing tailored support and could also be responsible for further referrals. A review found that, even when parents asked for additional help for their children, only one-third of them were helped by the child and adolescent mental health service, and that schools are important pathways to additional help [59].

In Norway there are a range of possible offers for CHIP, as economic support, for example for participating in spare time activities, time with an adult social contact, extra support from the teachers, help with homework, contact with the community nurse, time with another family during weekends or periods of time, treatment in child and adolescent mental service, and also inclusion of friends and family. Many of these offers for children are managed by the child welfare service.

The high referral rate to the child welfare services in our study could indicate that these options for prevention and help, most of them on a local level, were utilized as intended. However, parents wanted such help more than their children actually got, which could imply that the families/children’s needs are not appropriately detected. Referrals to the child welfare service in Norway are usually based on parental functioning.

Patients reported that nearly half the children were referred to the child welfare service, but only 14% of the health personnel confirmed this. This could be due to both parents and health personnel having difficulties talking about care issues, especially in the substance use services, and some parents being uncertain or fearful about referrals. Fear of talking about children’s needs can lead to children receiving less help than they need. A qualitative paper from substance use services found that health personnel had a tendency to choose either the children’s or the parents’ perspective [65].

### Factors correlated with contacts/referrals

Two parameters correlated with contacts/referrals: the children’s degree of mental symptomatology and the type of health service their parents received.

Children who suffered the most from mental health problems, as measured by the SDQ, were the subjects of the most contacts/referrals from the health personnel. This indicates that the parents’ health personnel were able to identify those children most in need of help, even if not to the degree desired by the parents.

The mental health and substance use services contacted/referred CHIP most often. This seems appropriate, as the literature suggests that these CHIP, in general, suffer the most distress [17–20]. It may also be that traditions for contacts/referrals in those services play a part here and that children whose parents are being treated in the physical health services may not be identified and helped according to their needs.

Several parameters were not associated with contacts/referrals. Studies of children of chronically ill patients have indicated that the youngest children and the oldest girls live with the most elevated risk [6]. Age did not turn out to be a factor related to referrals in our study. There are reasons to worry about the fact that the parents’ symptomatology and duration of symptoms did not play a part in our study, as the above-mentioned study found that those factors correlated with mental health problems for the children [6].

### The helpfulness of the legislation

A minority of the patients reported that the health personnel had informed them explicitly about the legislation. However, a majority had been asked if they had children, which implies that the health personnel were aware of the legislation. In line with this, it is understandable that a minority of patients reported that the legislation had been of help since they may not have been aware of the connection between the legislation and their being asked whether or not they had (minor) children. Only a few experienced the legislation as a burden, except for those receiving health services for substance use where 29.5% reported that the legislation felt like a burden.

This is a highly significant finding that requires further attention. People with substance use disorders, especially if they are parents, are often looked down upon and stigmatized [66–69]. Parents might face blame, a lack of respect and support that is shown to other patients. Creating a positive working alliance is, indeed, a special challenge for professionals in substance use services [65]. It is, therefore, important that health personnel have good communication skills that allow them to support patients when discussing difficult issues concerning their parenting and their children [56]. It is a challenge to keep a good working alliance with a patient with a substance use disorder, when pointing out that the patient’s child needs extra support, because of this. This may be a reason that patients with substance use perceive the legislation as a burden.

### Strengths and limitations

A strength of this study is its multi-centre design, as the five health authorities cover 34% of the Norwegian population and represent hospitals of different sizes as well as different socio-demographic areas. The inclusion of all fields of health care, i.e. physical health, mental health and substance use services, further broadens the picture and has provided new knowledge about the differences between the health service domains, as has the inclusion of different informant groups. Another strength of the study was that we managed to recruit a large number of participants.

A challenge of this study is that it is based partially on a questionnaire that has not been tested over time or validated. The questions about how health personnel comply with the legislation were developed by the study group. However, this did allow us to ask specific questions closely linked to the wording of the law, which would not have been achievable with any other questionnaire. The other questionnaires used for this study are widely used and validated.

The challenges of recruiting families living with illness are well-documented [70]. Our recruiters found it difficult to get patients and health personnel involved. We do not know the inclusion rate of families, as we did not receive reliable data on the number of patients who were informed about the study and invited to participate. Clinicians could have forgotten to inform about the study, or been reluctant to inform the patients and thus, we assume that a smaller portion agreed to participate.

Our ethical approval did not allow us to ask why patients did not want to participate. The recruiters’ impressions were that those patients struggling the most with their illness and everyday life were the most hesitant. It is possible that patients suffering from substance use were in a better state while hospitalized and interviewed for the study than they had been earlier. Moreover, many of them who had children did not have custody of them and, therefore, could not be included in the study. According to the recruiters, some health personnel did not inform their patients about the study because they considered their patients too vulnerable to be asked about their children. These factors taken together could have had a significant influence on how representative our group of informants was. It may be that the situation, in general, for families living with illness may be even more severe than our data indicate. One could ask whether we chose the most optimal recruiting procedure. The data are a bit dated but still interesting as they remain the only data broadly investigating Norwegian health personnel’s actual adherence to The Act. Therefore, the results of this study may be useful as a basis for further work on implementing new routines.

## Conclusions

The study found that, to some degree, health personnel met their new obligations in accordance with the health personnel legislation. However, children were not referred for help to the degree their parents wanted. They were most often referred to child welfare services, which was not what the patients most often preferred. This could reflect the ambivalent relationship between parents and this service.

Health personnel turned to a lesser extent to primary resources such as friends, family members, schools and community nurses, many of whom were already part of the children’s lives and could act in a preventive manner.

The substance use services referred children most often. Parents and health personnel reported quite different numbers in response to most of our questions. This underlines the importance of communicating effectively and clearly about children so that they can receive the appropriate help in a timely manner. A considerable number of parents who were being treated for substance use reported that the focus on the children had been difficult.

### Implications

Much more work must be done by health personnel in Norway, to secure contacts/referrals for CHIP in need for support/help. A reasonable implementation strategy was not elaborated in 2010. Implementation factors as leader involvement, economy, education, data systems and control systems are obviously needed for The Act to fill its full potential.

There is a potential for involving friends/family, the schools and public health nurses, especially from a preventive perspective. Appropriate communication between health personnel and ill parents is a necessary starting point for developing appropriate and timely help for CHIP. Not the least: children should be listened to. The substance use field is especially challenging. Careful and respectful conversations are needed to develop collaboration between parents and the services. Including several informant groups and several health care fields is appropriate when studying processes in the health care system in order to broaden the base for planning further appropriate help for CHIP.

## Data Availability

The data are stored at Akershus University Hospital. At this point they cannot be shared, as researchers are still working on the material. They can be delivered by the corresponding author on reasonable request.

## Abbreviations

CHIP: Children of Ill Patients
HSCL-90: Hopkins Symptom Checklist
SF-8: Health Survey Short Form
SDQ: Strength and Difficulties Questionnaire
HP: Health personnel
GP: General practitioner
SPSS: Statistical Package for the Social Sciences
Reg.no: Registration number
